# Occurrence, aetiology and challenges in the management of congestive heart failure in sub-Saharan Africa: experience of the Cardiac Centre in Shisong, Cameroon

**DOI:** 10.4314/pamj.v8i1.71059

**Published:** 2011-02-17

**Authors:** Jacques Cabral Tantchou Tchoumi, Jean Claude Ambassa, Samuel Kingue, Alessandro Giamberti, Sylvia Cirri, Alessandro Frigiola, Gianfranco Butera

**Affiliations:** 1 Cardiac Centre, St. Elizabeth Catholic General Hospital Shisong, Cameroon; 2 University of Yaoundé I, Faculty of biomedical sciences; 3 Department of Paediatric and Adult Cardiology and Cardiac Surgery, Policlinico San Donato IRCCS

**Keywords:** Congestive heart failure, cardiac centre Shisong, valvulopathies, cardiomyopathy, Cameroon, Hypertension, heart failure

## Abstract

**Introduction:** The aim of the study was to investigate the occurrence, the aetiology and the management of congestive heart failure in the cardiac centre of the St. Elizabeth catholic general hospital Shisong in Cameroon. **Methods:** Between November 2002 and November 2008, a population of 8121 patients was consulted in the referral cardiac centre of St. Elizabeth Catholic General Hospital. Of these patients, 462 were diagnosed with congestive heart failure according to the modified Framingham criteria for the diagnosis of heart failure. Complementary investigations used to confirm and establish the aetiology of the disease were the chest X-ray, electrocardiography, bi-dimensional Doppler echocardiography. **Results:** The results showed that the occurrence of congestive heart failure in our centre was 5.7%. Congestive heart failure was diagnosed in 198 females and 264 males, aged between 8 and 86 years old (42.5±18 years old). Post rheumatic valvulopathies (14.6%) and congenital heart diseases (1.9%) were the first aetiologic factor of congestive heart failure in the young, meanwhile cardiomyopathies (8.3%) in elderly followed by hypertensive cardiomyopathy (4.4%). Congestive heart failure was also seen in adults with congenital heart diseases in 0.01%. In this zone of Cameroon, we discovered that HIV cardiomyopathy (1.6%) and Cor pulmonale (8%) were represented, aetiological factors not mentioned in previous studies conducted in urban areas of Cameroon. The mean duration of hospital stay for the compensation treatment was thirteen days, ranging between 7 and 21 days), the mortality being 9.2%. All the medications recommended for the treatment of congestive heart failure are available in our centre but many patients are not compliant to the therapy or cannot afford them. Financial limitation is causing the exacerbation of the disease and premature death. **Conclusion:** Our data show a high incidence of congestive heart failure mainly due to post rheumatic valvulopathies in young patients in our centre. National program to fight against rheumatic fever and complications are of great urgency in our country. The compensation treatment of congestive heart failure is challenging in our milieu, characterized by poor compliance and financial limitations.

## Introduction

Heart failure is a major and growing public health problem around the world. Although published literature on heart failure in sub-saharan Africa is rare [1], there is evidence that the rate of hospital admissions for heart failure is comparable with rates to the rest of the world. However, the pathophysiology and aetiologies are different. In Africa, heart failure seems to be attributable mostly to systolic dysfunction and occurs as a major complication of high blood pressure. Heart failure represent the first cause of hospital admission among patients with high blood pressure in Africa. In internal medicine services, heart failure has been described as the fifth to sixth cause of admission [2,3]. There are no epidemiologic data on the prevalence of congestive heart failure (CHF) in rural Cameroon. Only few data are available in subsaharan Africa with studies conducted mostly in urban areas [4,5].

The cardiac centre is situated in a rural area of the North-West Cameroon where the population still rely on unprocessed biomass fuels in the form of wood, dung and crop residues. Furthermore, there is a very high incidence of HIV AIDS. The aim of this study was to investigate the occurrence and aetiology of CHF in the Cardiac Centre of St. Elizabeth catholic general hospital in Shisong, north-west Cameroon.

## Methods

**Patients and screening**

The study was approved by the Ethics Committee of St. Elizabeth Catholic General Hospital. Between November 2002 and November 2008, a population of 8121 patients was consulted in the referral cardiac center of St. Elizabeth Catholic General Hospital. Of these patients, 462 aged between 8 and 80 years (mean 42,5±18 years old) were diagnosed with CHF according to the modified Framingham criteria for the diagnosis of heart failure [6].The following criteria for heart failure were used.

**Major criteria:** Paroxysmal nocturnal dyspnoea raised jugular venous pressure, clinical cardiomegaly, basal crepitations, S3 gallop, clinical acute pulmonary oedema, pulmonary upper lobe blood diversion on chest X-ray (or pulmonary oedema on chest X-ray).

**Minor criteria:** tachycardia, orthopnoea, exertional dyspnoea, nocturnal cough, hepatomegaly, pleural effusion, diuretic use. CHF was diagnosed if the patient had two major and one minor or one major and two minor criteria. Severity of heart failure on admission was assessed using the NYHA functional classification. Detailed history including patients' socio-demographic characteristics, past medical history, and drug history were obtained from each patient through a standard questionnaire.

**Instrumental investigations**

Complementary investigations used to confirm and establish the aetiology of the disease were: chest X ray, electrocardiography, Trans-thoracic bi-dimensional Doppler echocardiography (TEE). TTE was performed according to the recommendations of the American Society of Cardiology using commercially available echocardiography equipment (Acuson Sequoia, Acuson Co, Mountain View USA) with a 4-7 Megahertz transducer. The TTE indices analysed included the left ventricle telesystolic diameter (LVIDS), left ventricle telediastolic diameter (LVIDD) using the parasternal long axis position in M-mode, and the M-mode ejection fraction (EF). Post rheumatic valvulopathy was defined by the presence of any definite evidence of mitral or aortic valve regurgitation seen in two planes by the TTE, accompanied by at least two of the following three morphologic abnormalities of the regurgitating valve: restricted leaflet mobility, focal or generalised valvular thickening, and abnormal subvalvular thickening [7]. Diastolic heart failure was diagnosed according to the recommendations of the American heart association [8].

**Data analysis**

Values are expressed as mean ± the standard deviation (SD) and statistical analysis were performed using the Student’s t-test. The SPSS 11 statistical analysis software was used for all analysis. The chi squared test was used to test for association between categorical variables. The student t-test was used to compare means of two variables, whilst the ANOVA was used to compare more than two variables. The level of significance was set at p < 0.05, and a 95 % confidence interval was applied to the numerical variables which are normally distributed.

## Results

**Aetiology of heart failure**

**Figure 1:**
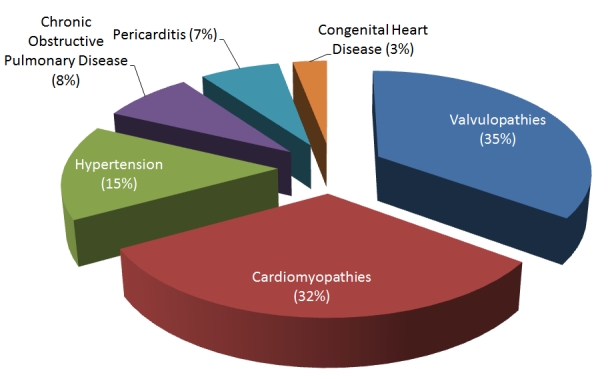
Aetiologic factors of heart failure in Saint Elizabeth Catholic General Hospital Shisong, cardiac center, Cameroon, from 2002 to 2008

The results showed that the occurrence of CHF in our centre was 5.7% while mortality from CHF was 9.2%. CHF was diagnosed in 198 females and 264 males, aged between 8 and 86 years (42.5±18 years old). CHF due to left ventricular systolic dysfunction was more prevalent (87.2%) than congestive heart failure due to diastolic dysfunction (12.8%), (p<0.05). Forty-four percent of patients were in class III of the NYHA and 7% in class IV. Dyspnoea was a constant symptom (95.2%) frequently associated to paroxystic nocturnal dyspnoea (68.1%). Lower limbs oedema and hepatomegaly were the most frequent physical findings (41.9%). In St. Elizabeth catholic general hospital Shisong, cardiac centre, congestive heart failure (Figure 1) is mainly due to valvulopathies (35%) and cardiomyopathies (32%) Other diagnoses were hypertension (15%), pericarditis (7%), chronic obstructive pulmonary disease (8%) and congenital heart diseases (3%). According to age (Table 1), post rheumatic valvulopathies, pericarditis and congenital heart diseases were the main aetiologic factor of CHF in pediatric patients. In adults, valvulopathies, cardiomyopathies and hypertension were the leading causes for CHF. In elderly (>60 years old) cardiomyopathies, hypertension and chronic obstructive pulmonary disease were more frequently diagnosed. The total mortality rate in the period of study was 9.2% recorded mostly in the group of young people with severe valvulopathy 42.3% and elderly with dilated cardiomyopathy 50.7%.

**Subgroups**

a) Post rheumatic mitral valve regurgitation was the pathology more encountered (Table 2); b)The more frequently diagnosed congenital heart disease was isolated ventricular septal defect 48.5%, patent arterial duct (18.7%). Other diagnoses were tetralogy of Fallot 29.1%, isolated atrial septal defect 3.2%; c) Cardiomyopathies are reported in (Table 3); idiopathic dilated cardiomyopathy was encountered in the large part of subjects; d) Pericarditis was mainly due to tuberculosis in HIV positive patients (83%).

**Instrumental assessment **

**Electrocardiography:** Left ventricular hypertrophy was the abnormality more frequently encountered (80%), followed by left auricular hypertrophy (31%) and right ventricular hypertrophies (15%). Left bundle branch block was seen in 27.7% of patients while right bundle branch block in 29.1%. Sinus rhythm was present in 62%, sinus tachycardia in 28% and atrial fibrillation in 10% of cases. Atrioventricular block I degree was also represented in few cases (7.2%). Myocardial infarction scar could be observed in 2.3% cases.

**Trans-thoracic Doppler echocardiography:** Patients with dilated cardiomyopathy showed diffuse dilatation of the four heart chambers and in particular of the left ventricle, the mean was 68.2 ± 2.5mm. Left ventricular function was significantly reduced (EF 32 ± 6%). Functional mitral valve regurgitation related to mitral valve annulus dilatation was frequently seen

In subjects with chronic obstructive disease of the lungs, the right atrium and ventricle were significantly dilated with associated moderate to severe tricuspid regurgitation and systolic pulmonary artery hypertension (mean estimated systolic pulmonary pressure 84.4±14mmHg, ranging between 70 and 114 mmHg).

Patients with post rheumatic valvulopathies showed fibrotic destruction of the mitral valve and aortic valves, calcified leaflets, abnormal subvalvular thickening rigid opening with coaptation default in patients with regurgitation and poor opening in patients with stenosis. In table 2 valvular findings are reported. Complications of rheumatic heart disease included secondary pulmonary hypertension in 11.8%, and functional tricuspid regurgitation was seen in 21.9% of patients.

In patients with hypertensive cardiomyopathy, we observed a concentric hypertrophy of the left ventricle with conserved systolic function but poor diastolic function.

**Hospital stay and treatment**

Forty-five percent of patients were coming from traditional healers. These subjects came frequently with skin infections after scarification, refractory lower limbs oedemas, prolonging their stay in the hospital. The main medications for the compensation treatment of congestive heart failure consisting of loop diuretics, angiotensin-converting enzyme inhibitor, digoxin, aldosterone inhibitors and beta blockers were prescribed in our centre as reported in literature. Being a caritative hospital, some of these medications are provided by non-government non-profit organisation from Europe and United States of America. The mean hospital stay for the compensation treatment was 13 ± 7 days (the range was7 to 21days). While admitted, all patients are receiving all medications recommended for the pathology. Pericardial tap in patients with tamponade was done with success in 78% cases. After the compensation treatment, patients with 25 patients with valvulopathies were operated upon in IRCCS Policlinico San Donato with the help of non-profit non-governmental organisations: Cuore Fratello and Associazione bambini cardiopatici nel mondo. Surgical total correction of congenital heart diseases was done in IRCCS Policlinico San Donato in 95 cases. In the newly open cardiac centre of Shisong, correction of congenital heart diseases was performed in mitral valve replacement was done in 22 cases surgical valvuloplasty was done in 8 cases, aortic valve replacement was performed in 6 cases.

**Follow-up**

In six years, there was loss of follow up 32.1%, late at follow up 40.1%. Loss at follow-up was related to financial limitations. Furthermore, the hospital is located far from main big Cameroonian cities and this situation discourages patients. Sometimes, because of the patient’s poverty, the cardiologist is obliged to choose among medications: instead of prescribing all the medication needed doctors have to prescribe just few drugs and more frequently loop diuretics, angiotensinconverting enzyme inhibitor, digoxine, potassium supplement. We call this way of prescribing “managing prescription”. Finally lack of finances causes the exacerbation of the disease and premature death.

## Discussion

Our results showed that the occurrence of congestive heart failure in St. Elizabeth catholic general hospital, cardiac centre is 5.7%, the mortality being 9.2%. In Spain, incidence of HF increased from 296 per 100,000 person-years in 2000 to 390 per 100,000 person-years in 2007 (RR 1.32, CI 95% 1.27-13.7, P<0.01) [9]. Although heart failure management has benefited from major advances in recent years, case fatality among people with heart failure remains high worldwide. In Brazil, the mortality is 6,3%, in Nigeria 4,3%[10,11]. Hospital death for CHF in Africa ranges from 9% to 12.5% [10]. This makes heart failure among the major causes of death of cardiovascular origin in Africa. The high mortality can be attributed to the lack of appropriate facilities and to disease complications on admission. In fact, most patients are admitted at NYHA class IV, in anasarca and shock. Kingue S et al showed in his study that more than 50% of patients were NYHA class III and IV[3]. Furthermore, many patients arrived at a late stage because of prior therapeutic attempts made by traditional healers. Many patients in rural areas believe more in traditional medicine “medicine of their ancestors” than in western medicine, reason why hospital is the last place they can be helped.

According to age, post rheumatic valvulopathies and congenital heart diseases are the first aetiological factor of CHF in young. Meanwhile dilated cardiomyopathies and hypertension were more frequently diagnosed in adults and elderly. In our experience, the causes of heart failure in subsaharan African remain largely nonischemic. However, coronarography was not available in our hospital during the time of the study. We believe that more cases of ischemic dilated cardiomyopathy could have been diagnosed by using an invasive approach.

In literature, it is reported that rheumatic heart disease, hypertension, chronic lung disease and pericardial disease are the main contributors to the aetiology of cardiac failure in sub-Saharan Africa, accounting for over 90% of cases [12]. Dominant aetiological factor of CHF in our milieu are post rheumatic valvulopathies. Our data are quite different from those reported by Mayosi BM in South Africa and by Onwuchekwa C and Asekomeh GE in urban Nigeria. Those authors reported an high incidence of hypertension as the dominant cause of heart failure [1,13]. This study according to Yusuf S et al [14] is showing this rural area is still in the phase of pestilence and famine characterised by a high rate of infectious diseases, low rate of men made diseases leading to cardiac problem.

In this study, HIV cardiomyopathy and cor pulmonale are represented as aetiologic factors in heart failure, not mentioned in previous studies conducted in urban areas of Cameroon and some countries of sub-saharan Africa [13,15,16], and mentionned in very high percentages by Magulaa NP and Mayosi BM [17,18] in studies carried out in south Africa. They stated that cardiac involvements in human immunodeficiency virus (HIV) infection, cor pulmonale, and pericarditis contribute to over 20% of cases of heart failure. Approximately half the world’s population and up to 90% of rural households in developing countries still rely on unprocessed biomass fuels in the form of wood, dung and crop residues here are high levels of air pollution, to which women, especially those responsible for cooking, and their young children, are most heavily exposed. In rural Cameroon particularly, the unprocessed biomass fuels are also used in houses for heating purposes [19,20].

Lessons from the changing epidemiology of heart failure in developed countries suggest that the burden of this disease will dramatically increase over the first half of this century [1]. Heart failure has emerged as an important form of cardiovascular disease in Africa. It has great social and economic relevance owing to its high prevalence, mortality and impact on young, economically active individuals. Concerning treatment three major trends emerge from few studies that have addressed the issue of management of heart failure: first of all underutilization of medications with proven efficacy such as ACEI and beta blockers when using the “managing prescription” because of poverty. For example, in a study from Nigeria, only 65% of heart failure patients were on ACEI [21]. In another study from Cameroon, 19% of patients with heart failure were on beta blockers. Second, when medications are appropriately prescribed, this is not always followed by patient compliance [22]. Finally, traditional treatments often delay diagnosis and proper therapy. Recent improvement in our centre with the construction of a fully equipped theatre and a cardiac catheterization laboratory for electric therapy and percutaneous treatment of heart diseases will increase the possibilities for heart diseases in our country [23].

## Conclusion 

Our data show a high incidence of congestive heart failure mainly due to post rheumatic valvulopathies in young patients in our centre. National program for fight against rheumatic fever and complications are of great urgency in our country. The compensation treatment of congestive heart failure is challenging in our milieu, characterized by poor compliance and financial limitation.

## Competing interests

The authors declared no one declared.

## Authors’ contribution 

ICMJE criteria for authorship met. All the authors have approved the final version of the manuscript.

## Acknowledgments 

The authors want to thank the congregation of the Tertiary Sisters of St Francis for their effort in the care of the patients, also the partners of the cardiac centre, Cuore Fratello, Associazione bambini cardiopatici nel mondo for their support and contribution in the realisation of the manuscript.

## Figures and Tables

**Table 1:** Aetiologic factors of congestive heart failure by age group, in the population of patient consulted at the referral cardiac center of St. Elizabeth Catholic General Hospital, Cameroon, from 2002 to 2008

**Age of patients**	**CHD**	**Valvulopathies**	**Cardiomyopathies**	**Hypertension**	**COPD**	**Pericarditis**
8-20 years old	1.9%	14.6%*	-	-	-	2.1%
21-40 years old	0.9%	9%	2%	2.3%	-	3.6%
41-60 years old	0.2%	6.4%	21.7%*	8.3%	2.5%	1.3%
61 years old and above	-	1%	8.3%*	4.4%*	5.5%	-

p<0.05; COPD: Chronic Obstructive Pulmonary Disease; CHD: Congenital Heart Disease

**Table 2:** Type of valvulopathies diagnosed Cameroon, from 2002 to 2008

**Type of the valvulopathy**	**Percentage**
Mitral valve regurgitation	59,7%
Mitral valve stenosis	26%
Mixed mitral valve disease	13,7%
Aortic valve regurgitation	15,3%
Aortic valve stenosis	7,2%
Combined aortic and mitral valvulopathy	5,2%

**Table 3:** Distribution of cardiomyopathies in St. Elizabeth catholic general hospital, cardiac centre, Cameroon, from 2002 to 2008

**Cardiomyopathies**	**Percentage**
Idiopathic dilated	90.2%
HIV associated	5.2%
Ischemic	3%
Hypertrophic	1.6%
